# Consensus on orthodontic treatment eligibility in Finnish health care system - A comparative analysis of assessment

**DOI:** 10.1093/ejo/cjaf060

**Published:** 2025-07-07

**Authors:** Annika Arpalahti, Anne-Maria Aulu, Elizabete Agafonova, Niina Raij, Heidi Arponen

**Affiliations:** Department of Oral and Maxillofacial Diseases, University of Helsinki, Po Box 41, Helsinki 00014, Finland; The Wellbeing County of Vantaa and Kerava, Myyrmäki Health Centre, PO Box 600, Vantaa 01088, Finland; The Wellbeing County of Vantaa and Kerava, Myyrmäki Health Centre, PO Box 600, Vantaa 01088, Finland; The Wellbeing County of Vantaa and Kerava, Myyrmäki Health Centre, PO Box 600, Vantaa 01088, Finland; The Wellbeing County of Vantaa and Kerava, Myyrmäki Health Centre, PO Box 600, Vantaa 01088, Finland; Department of Oral and Maxillofacial Diseases, University of Helsinki, Po Box 41, Helsinki 00014, Finland; The Wellbeing County of Vantaa and Kerava, Myyrmäki Health Centre, PO Box 600, Vantaa 01088, Finland

**Keywords:** orthodontics, public health care, treatment assessment, occlusal index, calibration

## Abstract

**Background:**

In many European countries, orthodontic treatment is offered in publicly funded healthcare to those with severe malocclusion. In the Finnish public health care, the modified Grainger’s treatment priority index (TPI) is used to determine malocclusion severity and treatment eligibility. The uniform use of occlusal indexes is crucial for equitable treatment allocation.

**Objective:**

This study aimed to investigate equity in access to orthodontic care in one wellbeing services county in Finland.

**Materials and methods:**

We conducted five calibration events, where five orthodontists were calibrated against one gold standard orthodontist with the longest clinical experience. All the orthodontists independently clinically examined random groups of patients using the Finnish modified TPI scoring. The malocclusion severity scores, diagnosis codes, and the determined treatment eligibility were subsequently compared. Agreement between the orthodontists was analyzed with Cohen’s Kappa and Bland-Altman statistics.

**Results:**

In total, 166 patients, aged 6–63 years, were examined across the calibrations, each representing a comparison between one of the five orthodontists and the gold standard. The agreement between the orthodontists was found to be substantial. The same judgement on treatment eligibility was made in 145 out of 166 cases (87%), whereas a different decision on treatment eligibility was made in 21 cases (13%).

**Conclusions:**

Despite the high level of interobserver agreement, differences in eligibility judgement were observed, which may have important consequences for the patient with respect to treatment provision. Therefore, systematic calibration and regular training in the use of national occlusal indexes should be implemented for all orthodontists.

## Introduction

Malocclusions are common, and may resolve spontaneously during dental development and growth [1]. Mild malocclusions do not always require corrective treatment [2]. The aim of orthodontic treatment is to normalize occlusal and dentofacial growth, and to correct dentofacial and orthognathic abnormalities [3]. The treatment is designed to maximize benefits for the patient while minimizing any possible harm. The extent and timing of the treatment is always evaluated on an individual basis [3].

In many European countries, orthodontic treatment is offered in a publicly funded system to individuals with a severe malocclusion [4]. Access to orthodontic treatment within public healthcare varies considerably across countries, reflecting differences in healthcare models, policy priorities, and funding mechanisms [4–8]. These disparities affect the availability of care; therefore, given the limitations of publicly funded resources, it is essential that their allocation is governed by principles of justice and equity. Occlusal indices guide the allocation of resources and treatment planning [9].

Existing literature outlines orthodontic practice policies and the use of indices in several European countries [10–13]. While some countries have integrated standardized tools, such as the Index of Orthodontic Treatment Need (IOTN) to determine treatment eligibility, others may lack standardized assessment protocols, affecting consistency in treatment provision [4]. The IOTN considers the nature and severity of a malocclusion, as well as the resulting esthetic impairment [14]. Most European countries prioritize orthodontic treatment within public healthcare for children and adolescents.

In the Finnish public healthcare system, the publicly funded orthodontic treatment of dentofacial abnormalities is offered free-of-charge to individuals under the age of 18 years, and at a lower cost than private practise to adults, according to the malocclusion criteria of the Ministry of Social Affairs and Health [5]. Emphasis is placed on malocclusions and abnormalities that might cause functional impairment or damage to long-term oral health. When the treatment criteria are met, the treatment is usually implemented in the public health centers or special health care units. Ministry guidelines state that all citizens should have equal opportunities for health services and freedom to choose the public health care unit in which they want to be treated, thus emphasizing the importance of concordant criteria for treatment [5].

The Finnish national criteria of non-urgent care in orthodontics are evaluated with a modified Grainger’s 10-point scale of treatment priority index (TPI) that is designed to objectively assess the severity of malocclusion and prioritize orthodontic treatment [5, 10, 15, 16]. In the developing occlusion, treatment is offered to those whose malocclusion score is 7 + or higher. In the fully developed occlusion treatment is planned when malocclusion scores are 9–10, or 8 when the malocclusion is considered to severely compromise oral health. In addition, orthodontic treatment is offered in cases where it is required for completing other dental treatment [5].

The 10-point TPI scale subdivides malocclusions as follows: Grades 1–4 represent a mild deviation from ideal occlusion, for which there is very little need for treatment, if at all [5]. Grade 5 is for a slight malocclusion with little treatment need. Grade 6 represents slight malocclusions with some need for treatment, and grade 7 represents malocclusions with moderate treatment need. 7 + refers to a moderate malocclusion with anticipated increasing treatment need. Grade 8 includes severe malocclusion with a great need for treatment, while grade 9 refers to a severe malocclusion with a very great need for treatment. Grade 10 includes craniofacial developmental disorders, such as cleft lip and palate. The TPI scale does not specifically consider esthetic or psychosocial factors. An orthodontist specialist usually determines the need and eligibility for orthodontic treatment in Finland.

Previous studies have noted a difference in the treatment eligibility decisions of orthodontist specialists [17, 18]. The differences are emphasized in borderline cases of moderate malocclusion [18, 19]. Such discrepancies are partly attributed to different interpretations of the criteria. For government funded orthodontics in the United Kingdom, calibrating education for dental professionals is offered to reduce discrepancies in the interpretation of the index [20, 21]. In Finland, both dental undergraduate and orthodontic postgraduate education includes teaching of the 10-point TPI-scale. However, systematic calibration is not performed or offered. Despite the observed differences in treatment decision percentages and evaluation of occlusal characteristics [10, 17], to our knowledge, no studies have addressed these differences over the past 20 years.

### Aims of the study

The aim of this calibration study was to investigate the conformity and equity in access to orthodontic care in the Finnish public health care system. Equitability, as an indicator of quality, was evaluated regarding treatment eligibility decisions to identify potential differences among orthodontic specialists at Wellbeing services county of Vantaa and Kerava.

The primary outcome measure was the discrepancy in the assessment of orthodontic treatment eligibility. The secondary outcome measure was deviation in evaluation of complexity of the malocclusion as assessed using the modified TPI. The study hypothesis was that orthodontist specialists similarly evaluate treatment eligibility in public healthcare.

## Materials and methods

This study was approved by the institutional research board of the Wellbeing County of Vantaa and Kerava. All participating orthodontists provided consent to participate in the study. Ethical approval was waived by the Institutional Research Committee.

For this study, six orthodontists in the Wellbeing County of Vantaa and Kerava conducted clinical examination of occlusion in pairs for random groups of consecutive patients with referrals from general dentists or dental hygienists for orthodontic screening. All the orthodontists had received similar training in the use of TPI during their post-graduate education. The orthodontist with the most extensive experience—13 years of clinical experience as a specialist orthodontist and a teaching position at the university—was designated as the gold standard, against whom all others were calibrated. During each clinical examination, the patient’s occlusion was visually assessed using a hand mirror and a ruler. Assessment conditions were standardized by employing a consistent examination procedure, uniform diagnostic tools, and systematic documentation of findings. For each pair of orthodontists, both independently evaluated the occlusion, graded the malocclusion with a 10-point scale, determined the diagnosis codes affecting their decision-making using the International Classification of Diseases (ICD-10) codes [22], and determined the ideal time for treatment onset. The orthodontists also had the option of not scoring a developing malocclusion, but instead either assigning the patient for follow-up or accepting the patient for an interceptive early-phase treatment to redirect abnormal growth or eliminate the developing malocclusion. The malocclusion scores, diagnosis codes and the determined timing of treatment onset/follow-up were subsequently compared.

### Statistics

Sample size calculation assumed that a difference in even one assessment of treatment need evaluation is clinically relevant and meaningful for the patient. To achieve 95% power with an anticipated malocclusion score difference of one and standard deviation of one, a minimum of 26 independent evaluations would be needed. The required sample size was determined based on the recommendations of previous publications, where 20–30 cases have been selected for calibration [21, 23–25].

For statistical analysis, the eligibility for treatment variable was dichotomized as either not eligibility or eligible for treatment or follow-up. Dental development was categorized according to age as early mixed dentition (5.6–9.4 years), late mixed dentition (9.5–12.4 years) or permanent dentition following the reported usual eruption timing [26]. The association between agreement in evaluated treatment eligibility, dental development phase, and primary malocclusion diagnosis (i.e. the diagnosis that was the most serious and decisive) was evaluated across the calibrations with a Chi-square test and Fisher-Freeman-Halton exact test, respectively, to determine whether these patient characteristics influence uniform decision-making.

Cohen’s Kappa was calculated to quantify inter-rater agreement in each calibration event, where every calibration represented a comparison between one of the five orthodontists and the established gold standard. A Bland-Altman analysis was conducted to evaluate the level of agreement and potential systematic bias between the orthodontists. SPSS statistics 29.0.2.0 (20) was used for analysis. Statistical significance was set at 0.05.

## Results

In total, 166 patients, aged 6–63 years, were examined across the five calibration events (Table 1). Of the patients examined, 64%–82% were deemed to qualify for publicly funded orthodontics.

**Table 1:** Calibration of evaluation of orthodontic treatment need between six orthodontists on five different occasions.

Orthodontist^*^	Years of experience^**^	Patients’ age range/years	Patients N	Admitted to treatment	Scheduled for treatment or follow-up/N (%)	Kappa measure of agreement (Confidence interval)	p-value
1	1	7.3–17.7	39	12	27 (69)	0.77 (CI 0.54–0.95)	<0.001
2	2			14	25 (64)		
1	1	7.3–16.1	31	8	23 (74)	0.84 (CI 0.58–1.0)	<0.001
3	2			10	21 (68)		
1	1	6.0–17.6	32	7	25 (78)	0.55 (CI 0.21–0.85)	0.001
4	1			11	21 (66)		
1	1	7.67–21	34	6	28 (82)	0.53 (CI 0.10–0.82)	0.002
5	2			7	27 (82)		
1	1	7.3–63	30	10	20 (67)	0.62 (CI 0.30–0.89)	<0.001
6	2			9	21 (70)		

^*^1 = gold standard orthodontist, 2–6 = other orthodontists.

^**^1 = three or more years in practice, 2 = less than three years in practice.

When considering all calibration occasions collectively, the combined results indicate that the same judgement on treatment eligibility, independently evaluated by pairs of orthodontists, was made in 145 out of 166 cases (87%), whereas a different decision on treatment eligibility was made in 21 cases (13%). The statistically moderate to substantial level of agreement between the orthodontists were reflected in high Kappa coefficient scores (Table 1).

The results of the Chi-Square test indicated that there was no significant association between the agreement on treatment eligibility by the orthodontists and the age of the patient (p = 0.617). The crosstabulation showed that among the cases where the treatment eligibility decision was made and the same judgement on treatment eligibility was presented, 39% of patients were in the early mixed dentition phase, 41% of patients were in the late mixed dentition phase, and 20% of patients had complete dental development. On the other hand, when a different judgement on treatment eligibility was presented, 33% of patients were in the early mixed dentition phase, 52% in the late mixed dentition phase, and 14% in fully developed dentition. The percentage of patients scheduled for treatment or follow-up by an orthodontist was not associated with years of professional experience (p = 0.694) (Table 1). The Fisher-Freeman-Halton exact test showed a statistically significant difference between the agreement on treatment eligibility and the primary diagnosis of the patient (p = 0.035). Of the 145 cases with the same opinion on treatment eligibility, crossbite (34%) and crowding (30%) were the most prevalent primary diagnosis. Of the patients with a primary diagnosis of crossbite, 85% of the evaluations resulted in the same judgement by both orthodontists. A high prevalence of agreement on treatment eligibility decision was also found in patients diagnosed with scissors bite (83%). The same judgement by both orthodontists was the least common in patients with a primary diagnosis of distal occlusion (28%), crowding (46%), and deep bite (48%). Of the 21 cases with differing opinions, crowding and deep bite were the most common primary diagnosis (29% each), and open bite was the third most common (19%). Table 2 outlines the frequency distribution of interobserver agreement across all registered diagnosis. These results suggest that, in our sample, the disagreement between orthodontists on definite treatment eligibility seems to be unrelated to the patient’s age, as reflected by dental development phase. However, differences in treatment eligibility decisions were significantly more common in patients with vertical malocclusion.

**Table 2.** Distribution of agreement/no agreement on treatment eligibility according to malocclusion traits observed. Each patient (N = 166) has one to three diagnosis codes.

	Diagnosis	Crossbite		Crowding		Deep bite		Open bite		Distal occlusion		Large overjet		Mandibular retrognathism		Mandibular prognathism		Hypodontia		Spacing		Scissors bite		Ectopic eruption		Total number of diagnosis	Percentage of agreement
	Agreement	yes	no	yes	no	yes	no	yes	no	yes	no	yes	no	yes	no	yes	no	yes	no	yes	no	yes	no	yes	no		
Calibration between orthodontist	1 and 2 (39 patients)	8	0	10	1	4	3	2	1	3	0	2	1	2	0	2	0	2	0	1	0	1	0	1	0	44	86
	1 and 3 (31 patients)	6	1	9	2	4	0	3	2	1	0	4	0	0	0	1	0	5	0	0	0	3	0	0	0	41	88
	1 and 4 (32 patients)	6	1	6	3	3	3	3	0	1	0	5	0	0	0	0	0	1	0	0	0	1	0	1	0	34	79
	1 and 5 (34 patients)	10	0	7	2	14	1	1	0	1	0	5	0	0	0	1	0	2	1	0	0	5	0	1	0	51	94
	1 and 6 (30 patients)	10	0	10	0	5	2	2	1	0	0	2	2	0	0	0	0	3	1	0	0	2	0	1	1	42	81
	Total	41	1	43	7	29	10	11	4	6	0	18	3	2	0	4	0	13	2	1	0	12	0	4	1		

**Figure 1.**
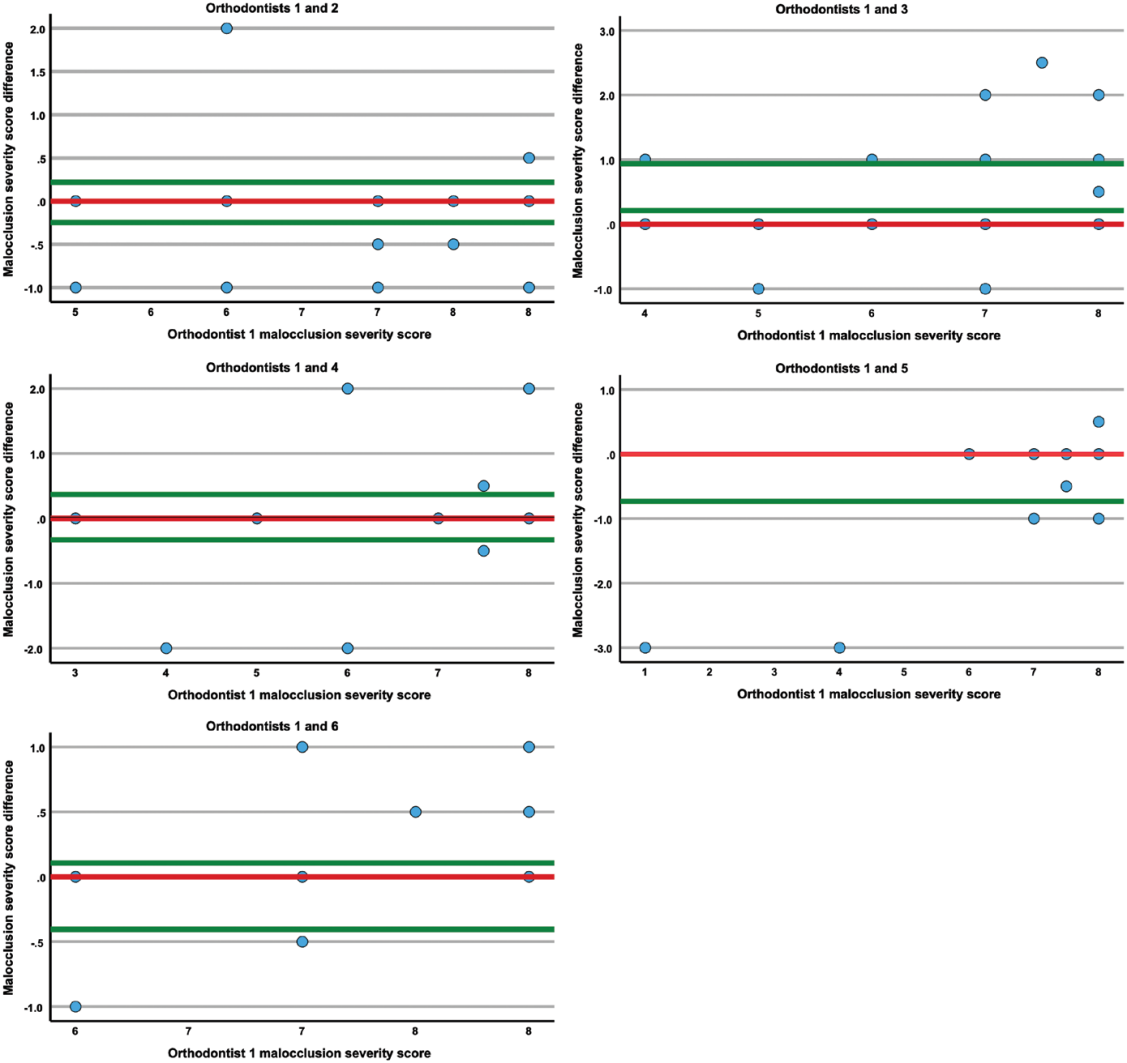
The difference in malocclusion severity score assessments (modified Treatment Priority Index) of the orthodontists relative to the reference value by orthodontist 1 (gold standard). a. Orthodontists 1 and 2. b. Orthodontists 1 and 3. c. Orthodontists 1 and 4. d. Orthodontists 1 and 5. e. Orthodontists 1 and 6.

A Bland-Altman analysis to evaluate the agreement between orthodontists revealed that the mean interobserver difference between the malocclusion severity scoring of orthodontist 1 (gold standard) and orthodontist 2 was −0.015 (95% CI [−0.248, 0.219], n = 34) (Fig. 1). The distribution of discrepancies indicated a systematic bias where orthodontist 2 consistently assessed malocclusion as more severe than orthodontist 1. However, the magnitude of the difference was one point at maximum and would have been clinically significant for just one patient (3%), who was deemed eligible for treatment by one orthodontist but not the other.

The mean difference between the malocclusion scoring of orthodontist 3 compared with orthodontist 1 was: 0.574 (95% CI [0.211, 0.937], n = 27), indicating a slight systematic bias where orthodontist 3 frequently evaluated malocclusion to be less severe than the gold standard. The difference in scoring resulted in clinically significant differences in treatment decision for one patient (4%).

Similarly, the mean difference in malocclusion severity scoring of orthodontist 4 against orthodontist 1 was 0.020 (95% CI [−0.330, 0.370], n = 25), indicating no proportional bias. The difference in scoring resulted in clinically significant differences in decision regarding three patients (12%).

The mean difference in malocclusion severity scoring of orthodontist 5 compared to orthodontist 1 was −0.354 (95% CI [−0.725, 0.017], n = 24), indicating a slight systematic bias where orthodontist 5 frequently assessed malocclusion to be more severe than gold standard. The difference in scoring resulted in clinically significant differences in decision regarding one patient (4%).

The mean difference in malocclusion severity scoring of orthodontist 6 compared to orthodontist 1 was −0.146 (95% CI [−0.400, 0.107], n = 24), indicating a random difference. The difference in scoring resulted in clinically significant differences in treatment decision for three patients (13%).

## Discussion

In many European countries, the government covers orthodontic treatment, at least partially, provided that set criteria are fulfilled. Occlusal indices are used extensively not only in determining access to public health orthodontics or the level of third-party copayment, but also in quality assurance and research [27, 28]. This study found a substantial level of agreement among the six orthodontists independently assessing treatment eligibility based on the national malocclusion evaluation index. However, differences in decisions on treatment eligibility were detected in 6%–21% of cases. Importantly, a difference in treatment eligibility decision is significant for the individual patient.

The IOTN and its modifications are well established in many European countries [13, 14]. IOTN ranks malocclusion based on the significance of various occlusal traits for dental health and aesthetic impairment, and has been shown to be both valid and reliably reproducible [14, 27]. The Dental Health Component (DHC) of the IOTN is an adaptation of the Swedish Medical Health Board index [29–31].

In the United Kingdom, orthodontic assessments under the NHS for individuals under 18 years are generally made using the DHC of the IOTN [32]. In the primary care setting, patients with IOTN DHC 3.6 or higher are selected for treatment. In the secondary care setting, the IOTN DHC is usually required to be at least 4 [8, 32]. The difficulty of treatment is also taken into consideration when deciding between primary and secondary care [8]. Treatment is offered to those who benefit from the treatment in terms of oral health and/or psychosocial well-being [8, 32]. In Germany, Orthodontic Indication Groups (KIG) is a diagnosis-based classification system based on the IOTN, and is used to determine whether orthodontic treatment costs of private clinics are reimbursed under statutory health insurance [11]. Malocclusions are categorized into eleven etiological groups and assigned to one of five degrees of severity [11, 33]. A similar modified IOTN system of classification is practiced in Scotland and Ireland [4].

In Norway, the public funding subsidy depends on the severity of the malocclusion [7]. The need for orthodontic treatment is divided into four categories [12]. In the ‘very great need’ category, the treatment is 100% funded, while in the ‘great need’ category, the treatment is 75% funded, and in the ‘obvious need’ category, public funding covers 40% [7]. Notably, treatment costs are partly or fully covered only in patients under 18 years of age [7]. Similarly, in Iceland, orthodontic treatment is partly subsidized if it was initiated before the individual turns 21 and set criteria are met [34]. Severe malocclusions related to congenital defects, diseases, or accidents can be covered for up to 95% of the treatment costs [34]. The Swedish public health care system offers full funding until the age of 19 years when a malocclusion is classified as requiring urgent or very urgent treatment need on a 5-grade treatment priority index [6, 31, 35]. In parallel, in Denmark, those with ‘moderate or major malocclusion’ are referred to an orthodontist for assessment. According to Danish Ministry of Health and Prevention, the treatment is offered free-of-charge until the age of 18 years^.^if the malocclusion ‘imply a foreseeable or already existing risk of physical damage or psychosocial strain’ [18, 34].

Treatment priority index (TPI), on which the Finnish index system is based, was developed to assess the severity of the most common types of malocclusion, the degree of handicaps they cause, and priority of treatment [15]. The index consists of eleven weighed and defined measurements and seven malocclusion syndromes (i.e. conditions). The TPI was designed to assess developing occlusions and mixed dentition, whereas the IOTN was originally developed for late mixed dentition and permanent dentition [14, 15]. Most orthodontists in the UK are calibrated in the use of the DHC of the IOTN [21, 32]. However, previous reports have concluded that dental practitioners in the UK show subjective agreement when determining orthodontic treatment need [36]. In the Netherlands, a calibration study applying the Index of Complexity, Outcome and Need (ICON) found that inter-examiner agreement was moderate to good for non-calibrated orthodontists, and was good for calibrated orthodontists [37]. To our knowledge, evidence regarding inter-orthodontist or dentist agreement in applying other indices besides IOTN is scarce, despite the clinical significance. In general, practitioners’ subjective assessments of treatment need are found to be only moderately reliable overall [36]. Previous studies examining the agreement between practitioners and consistency in evaluation of treatment need highlight the variability in assessments and the importance of standardized indices such as the IOTN and Peer Assessment Rating (PAR) [38]. It was noted that the assessment method influences treatment need decision [38]. In contrast to the UK, where dentists are calibrated in the use of the IOTN index, systematic calibration for the 10-point scale TPI is not done in Finland. This can be problematic with respect to equal rights to qualify for treatment regardless of where the individual lives or where they choose to get treatment from. In the current study, we had one gold standard orthodontist against whom five other orthodontists were calibrated in a real clinical setting, which is a strength of the study. However, the use of five different patient groups represents a weakness. In addition, intra-examiner error was not evaluated for the gold standard orthodontist. Nevertheless, the number of patients in each group fulfilled the number determined in the sample size calculation. Studies on larger samples could reduce potential case-related and methodological confounders.

The percentage of patients selected for orthodontic treatment was reasonably high, 65%–82%, due to the referral system for orthodontic evaluation. All the patients were prescreened by a general dentist or dental hygienist, and only those considered eligible for orthodontic treatment were referred. For that reason, the number of so-called borderline cases was probably higher than if the patients had been selected from the age cohort at random. Reaching a consensus is easier in extreme cases, such as those that are either very mild or very severe.

Although there were differences in scoring of the malocclusions between orthodontists, and systematic bias was found between orthodontists 1 and 2, 1 and 3 and 1 and 5, this was clinically significant in only 9 patients out of 166 (5%). However, even random differences in treatment eligibility decision-making between professionals may lead to meaningful consequences for an individual patient. Orthodontist 2 consistently assessed malocclusions to be more severe than the gold standard, orthodontist 3 frequently assessed them to be less severe than the gold standard. Thus, orthodontists 2 and 3 appear to stand on different ends of the spectrum considering the points given to malocclusions. This emphasizes the need for systematic calibration and regular training of the occlusal indexes used to ensure consistent and fair decision-making.

In this study, only part of the patients selected for public treatment were scheduled for treatment right away. Other treatments were to be started later, or patients were to be followed up for different reasons. We can predict that some of the patients who were designated for a follow-up would possibly not qualify for later treatment if the malocclusion severity would be reduced during growth. The opposite is also possible in the Finnish health care system. The children are examined several times during their growth period by a general practitioner or dental hygienist, and a new referral to orthodontic screening is possible if the malocclusion severity increases. Our findings suggest that transverse malocclusions were consistently diagnosed and deemed to require treatment by the orthodontists, whereas vertical malocclusions were not identified as requiring treatment in a uniform way. Crowding was a prevalent primary diagnosis both among those patients deemed to need treatment and among those not eligible for treatment. This finding is likely explained by the varying degree of crowding and the evolving dental development phase, which makes prediction of malocclusion development challenging. Without a longitudinal follow-up study, it is impossible to determine how many patients would have had a different malocclusion treatment outcome by the time they reach adulthood.

## Conclusions

Although interobserver agreement was generally high, variations in judgment were still present, which could affect the patients’ entitlement to publicly funded orthodontic treatment. Therefore, based on the findings of this study, we conclude that in public health care, standardized indices should be applied, and orthodontists should be calibrated for the use of national treatment need index. In addition, regular training updates should be offered to maintain consistency in applying the index. Implementing structured assessment tools and regular calibration exercises can help reduce subjective biases and enhance the reliability of treatment need evaluation.

## Data Availability

All data generated or analyzed during this study are included in this article. Further enquiries can be directed to the corresponding author.
